# Overlap Syndrome in Late-Onset Systemic Lupus Erythematosus With Lupus Nephritis and MPO-ANCA Pauci-Immune Glomerulonephritis and Tuberculosis: An Uncommon Association

**DOI:** 10.1155/crin/5285961

**Published:** 2025-02-26

**Authors:** Jaime Arturo Dulce Muñoz, Gustavo José Aroca Martínez, Christian David Seni Hernández, Diana Marcela Perea Rojas, Omar Cabarcas Barbosa, Lucia Mercedes Niño Hernández, Dario Jose Gaivao Arciniegas, Camila María García Jarava, Marianela Olivares Olmos, Sebastian Andre Seni Hernández, Valentina Pérez Jiménez, Indiana Luz Rojas Torres

**Affiliations:** ^1^Nephrology Department, De La Costa Clinic, Barranquilla, Colombia; ^2^Faculty of Health Sciences, Simon Bolivar University, Barranquilla, Colombia; ^3^Faculty of Health Sciences, Del Norte University, Barranquilla, Colombia

**Keywords:** ANCA, ANCA-associated vasculitis, antibodies, antineutrophil cytoplasmic, glomerulonephritis, lupus erythematosus, lupus nephritis, MPO, myeloperoxidase, systemic, vasculitis

## Abstract

Systemic lupus erythematosus is a systemic autoimmune pathology that generally presents in young people and manifests acutely, while its late presentation in people over 50 years of age is rare and insidious. Vasculitis is a pathology that affects any vessel producing fibrinoid necrosis, and presents with a positive antineutrophil cytoplasmic antibody. The concomitance of these two entities is rare and leads to worse clinical outcomes. We present a 73-year-old female patient who presented with rapidly progressive glomerulonephritis requiring renal replacement therapy, pulmonary tuberculosis, late-onset lupus erythematosus with lupus nephritis, and a positive result for neutrophil cytoplasmic antibody. An immune-mediated extracapillary proliferative glomerulonephritis was found when the biopsy was performed, with obvious signs of vasculitis, an overlap syndrome was found between these entities. She was initially treated with antituberculosis therapy, boluses of methylprednisolone and continued with intermittent renal replacement therapy; however, due to the severity of his pathologies, she had a fatal outcome. The concomitance between these autoimmune pathologies is unusual; there is a late-onset overlap syndrome between lupus nephritis accompanied by myeloperoxidase-antineutrophil cytoplasmic antibody and pauci-immune glomerulonephritis. The dual presentation establishes clinical challenges for its diagnosis as well as the initiation of immunosuppressive therapy when there are additional infectious pathologies.

## 1. Introduction

Systemic lupus erythematosus (SLE) is an autoimmune rheumatological disease with multisystemic involvement characterized by the production of antibodies, of uncertain etiology, with a higher prevalence in women of reproductive age with a worldwide annual incidence rate between 1 and 10 per 1,00,000 habitants and a prevalence of 20–70 per 1,00,000 habitants [1, 2]. In Colombia, there were 41,804 cases of SLE reported in 2016, indicating an estimated prevalence of approximately 91.9 cases per 1,00,000 individuals [3, 4]. Regarding vasculitis in Colombia, Takayasu's arteritis is the most frequent in accordance with previous reports from Latin American countries [5, 6].

SLE diagnosis is based on clinical and paraclinical criteria according to EULAR/ACR (European League Against Rheumatism/American College of Rheumatology), where patients must have a minimum of 10 points to make the diagnosis [7]. Late-onset SLE occurs in patient between 60 and 70 years old, is rare and uncommon, accounting for 2%–12% of all SLE patients worldwide [8, 9] and less defined clinical manifestations are usually present compared to those found in the fertile population, it has been shown that it is more frequent to find anti-Ro in 90%, and anti-La in 60%, and anti-DNA in 30% [10].

Lupus nephritis (LN) is the renal involvement of SLE, characterized by the presence of proteinuria in 24 h ≥ 500 mg/dL, active sediment in urine analysis, presence of ANA and/or anti-DNA, and its correlated with renal biopsy [2].

On the other hand, vasculitis is inflammation of blood vessel walls, and blood vessels of varying type and in different organs may be affected, thus the signs and symptoms of disease are extremely diverse and may overlap with many other diseases [10, 11].

We present a 73-year-old female with rapidly progressive glomerulonephritis, pulmonary tuberculosis, late-onset lupus erythematosus with LN, and positive neutrophil cytoplasmic antibody. Despite treatment with antituberculosis therapy, methylprednisolone, and intermittent renal replacement therapy, she had a fatal outcome. The dual presentation posed significant diagnostic and treatment challenges.

### 1.1. Case Presentation

The case involves a female 73-year-old patient, retired, from Barranquilla (Colombia), with a history of left nephrolithiasis, who consulted on 18/April/2024 at her primary care center due to a clinical picture of 48 h of evolution characterized by episodes of bilious vomiting, accompanied by altered level of consciousness and lower limbs edema. Therefore, dialysis urgency was identified and renal replacement therapy was initiated. Then, the patient was referred to our institution for specialized medical attention.

**Figure 1:**
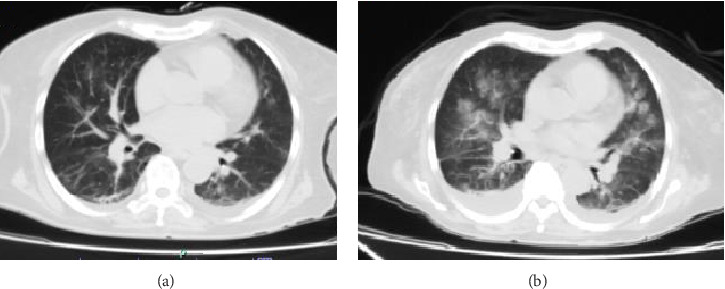
Chest computed tomography scan. The figure shows multiple perihilar infiltrates bilaterally, along with evidence of a predominantly right-sided pleural effusion (a) and bilateral perihilar alveolar compromise and bilateral basal pleural effusion, with an infectious component (b). Source: patient's medical chart, 2024.

Upon admission, the patient was in regular overall condition, receiving oxygen via nasal cannula with low oxygen saturation levels. Physical examination revealed grade I edema in the lower limbs and signs of pulmonary congestion. Additionally, initial laboratory tests showed mild hypoxemia, grade II anemia without transfusion requirement, and elevated azotemia without urgent criteria. Furthermore, a simple chest computed tomography (CT) scan revealed multiple perihilar infiltrates bilaterally, along with evidence of a predominantly right-sided pleural effusion (Figure 1(a)).

In addition to these findings, the patient presented with progressive alopecia, asymmetric arthralgias involving the extremities, and signs of inflammatory synovitis in the hands and right shoulder. The patient also reported a history of nonquantified fever. Combined with an evident autoimmune profile, these findings align with the EULAR/ACR classification criteria for SLE, with the patient accumulating 21 points under these criteria.

**Figure 2:**
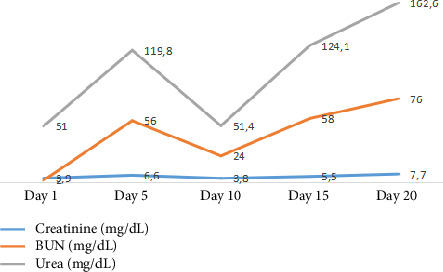
Evolution of paraclinical tests during the hospital stay. The figure shows evolution of renal function tests during the hospital stay. Source: patient's medical chart, 2024.

Therefore, due to the findings of congestion, treatment with a diuretic was initiated; however, the patient exhibited a regular respiratory pattern, with reports of elevated azotemia levels, and urinary output of 0 cc/kg/hr over 12 h despite an increase in the dose of intravenous diuretic (see Figure 2). Consequently, the patient was transferred to the intensive care unit (ICU) due to a high risk of hemodynamic deterioration, and renal replacement therapy was initiated in conventional intermittent mode with right femoral vascular access.

A second chest CT scan was performed, revealing bilateral perihilar alveolar compromise and bilateral basal pleural effusion, with an infectious component (see Figure 1(b)). The patient was re-interviewed and reported weight loss in recent months and hemoptysis. Consequently, fibrobronchoscopy with bronchoalveolar lavage was performed, along with culture and genexpert testing for *Mycobacterium tuberculosis*, which returned positive for drug-sensitive tuberculosis. Thus, antitubercular treatment was initiated, adjusted according to renal function, with ethambutol 400 mg (2 tablets orally every 48 h), pyrazinamide 500 mg (3 tablets orally every 48 h), and rifampicin 150 mg + isoniazid 75 mg (4 tablets orally every 24 h, omitting Sundays).

Additionally, an immunological profile was carried out, reporting hypocomplementemia, and positive AntiDNAn and ANAs, thus, the patient was contextualized with a SLE and treatment with hydroxychloroquine was initiated. A renal biopsy was performed, reporting a proliferative extracapillary glomerulonephritis with immune-mediated deposits of IgM and C3 both in the tufts and in the crescents. Subsequently, an autoimmune profile was obtained with a positive result for MPO-ANCA and negative for antiglomerular basement membrane antibodies (see Table 1).

**Figure 3:**
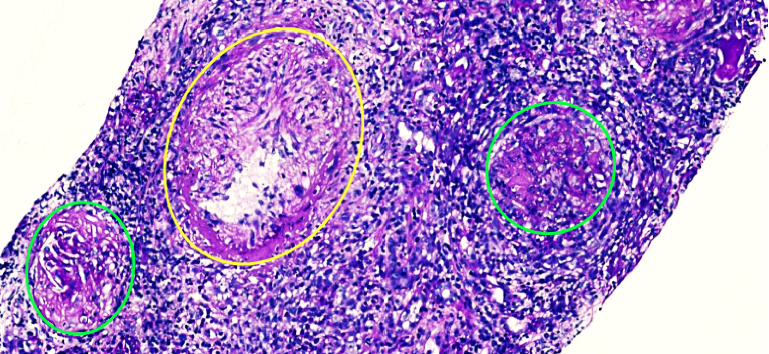
Renal biopsy. The figure shows collapsed glomerulus (green circle), an edematous vessel (yellow circle), and dense inflammatory infiltrate of lymphocytes. Source: patient's medical chart, 2024.

Additional findings include extensive necrosis, severe inflammatory reaction, periglomerular granulomas, and the presence of an intermediate vessel with an arteritis process (Figure 3). Based on the aforementioned findings and the patient's context of SLE, the condition was considered as LN and therefore, methylprednisolone was initiated at a dose of 500 mg intravenously for 3 days continuing with antituberculosis treatment, however, her acute respiratory infectious process limited the treatment regimen and cyclophosphamide was not administered. Unfortunately, the patient developed infectious complications and had a fatal outcome.

## 2. Discussion

SLE is the prototype of an autoimmune disease that can affect multiple organ systems, leading to diverse manifestations and clinical outcomes. The development of this pathology involves a complex interplay of environmental factors, toxins, infections, genetics, and sex [12].

The most common presentation is early-onset SLE, which accounts for up to 80% of cases, while late-onset SLE ranges from 2% to 20% of cases. LN is present in 40% of SLE patients and can lead to significant morbidity and mortality if the outcomes are compromised. Many studies have found that the late-onset course of SLE is typically more insidious and less aggressive. However, the case presented here demonstrates an acute onset with severe involvement, particularly in the renal component, manifesting as rapidly progressive glomerulonephritis [13, 14].

This is likely due to several factors, including the presence of a crescentic glomerulonephritis pattern, which is associated with a worse renal prognosis and higher mortality. Importantly, this case also had an associated MPO-ANCA vasculitis, a rare concomitant condition. The diagnosis of an “overlap syndrome” was made, as the patient met the classification criteria for both SLE (21 EULAR/ACR points) and MPO-ANCA vasculitis based on the positive serological findings and histopathological evidence of medium-vessel vasculitis with fibrinoid necrosis [10, 11].

The renal biopsy analysis revealed a pattern consistent with extracapillary proliferative glomerulonephritis, characterized by endocapillary hypercellularity, diffuse crescent formation, and periglomerular granulomas. Immunohistochemistry demonstrated positivity for C1q and low-intensity IgM deposition in the membrane and crescents. Additionally, a medium-sized vessel showed evidence of vasculitis with fibrinoid necrosis. The findings were negative for tubular necrosis, extraglomerular interstitial granulomas, or microorganisms suggestive of granulomatous tubulointerstitial nephritis due to tuberculosis. The Ziehl-Neelsen stain was also negative for membranous patterns. These observations suggest possible synchronism of pauci-immune glomerulonephritis with concomitant immune-mediated glomerulonephritis. This dual pattern may account for the atypical findings in this case.

In addition to the findings related to LN, the renal biopsy also revealed significant signs of vasculitis and medium vessel arteritis. The observed crescentic injury pattern, associated with nephritic syndrome, is noteworthy. Paraclinical studies confirmed positive results for P-ANCA and MPO-ANCA, which strongly suggests a high likelihood of microscopic polyangiitis. Based on these findings, the patient was assigned a score of six points according to the EULAR criteria, reinforcing the diagnosis of autoimmune-mediated kidney involvement. These results are crucial to consider, as they could suggest a possible overlap of LN with a vasculitic component, which may impact the patient's prognosis and therapeutic management.

The fatal outcome in this case highlights the aggressive nature of this association, which has been described in several previous reports. It is important to consider that infectious processes and medications can trigger the development of these pathologies in susceptible individuals. In this case, the *M. Tuberculosis* infection likely played a significant role in the pathophysiology [15].

The presence of ANCA antibodies in patients with SLE has important clinical implications. Firstly, ANCA, especially MPO-ANCA, are associated with higher disease activity and more severe clinical manifestations, such as LN [16]. Several studies have shown that patients with SLE who test positive for ANCA have a higher incidence of LN, as well as elevated levels of anti-dsDNA antibodies and a decrease in complement levels (C3 and C4) [17, 18]. Additionally, the presence of ANCA in SLE patients may be related to specific histopathological features in the renal biopsy, such as a higher prevalence of glomerular necrosis and crescent formation. This suggests a pattern of necrotizing glomerular inflammation similar to that observed in ANCA-associated vasculitis. These characteristics may influence renal prognosis, as ANCA-positive patients tend to have more impaired baseline renal function and higher serological activity of lupus [19].

Given the severity of the condition, aggressive immunosuppressive treatment was essential, but the initiation of methylprednisolone boluses was delayed due to the acute pulmonary tuberculosis, which restricted the therapeutic options. In regions with a high frequency of tuberculosis, some recommendations suggest prophylactic isoniazid treatment, regardless of the tuberculin test result, but there is no clear consensus on the optimal timing for initiating immunosuppression. The decision to initiate it in patients with autoimmune diseases who present with concomitant infections represents a clinical challenge. On the one hand, it is necessary to control the activity of the underlying disease to avoid complications. However, the administration of immunosuppressive drugs could exacerbate the infectious process and further compromise the patient's health status. The available evidence is limited to isolated case reports and small patient series. There are no clinical practice guidelines or recommendations based on controlled studies to guide management in these situations. Each case must be evaluated individually, considering factors such as the severity of the infection, the degree of activity of the autoimmune disease, and the patient's comorbidities.

In general, it is recommended to first treat the infection with appropriate antimicrobials and delay the initiation of immunosuppressants until control of the infectious process. However, in patients at risk of irreversible organ damage due to autoimmune disease activity, cautious use of corticosteroids or other low-dose immunosuppressive agents, in conjunction with antibiotics or antivirals, may be justified. More prospective studies and patient registries are needed to establish evidence-based management guidelines. In the meantime, decisions must be made on an individualized basis, weighing risks and benefits, and closely monitoring the patient.

In the presented case, it was taken to a medical meeting deciding to establish antiphymic therapy for a month and then start cyclophosphamide. In conclusion, the concomitance of SLE and vasculitis should be suspected, and active investigation for vasculitis is crucial, even though false-positive ANCA results can occur [20, 21]. Treatment should be tailored to the predominant pathology, and a negative ANCA serology does not exclude the possibility of vasculitis if the histopathological findings are present [22, 23].

## Figures and Tables

**Table 1:** Autoimmune and infectious profile.

Test	Result	Interpretation
VDRL	Not reactive	Negative
HIV	Not reactive	Negative
HBsAg	1.3	Negative
CMV IgG	0.37	Negative
CMV IgM	0.63	Negative
EB IgG	4.63	Positive
EB IgM	0.01	Negative
ANTI SM	< 4.9	Negative
ANAS	1/640	Positive
ANTI RO	< 3.3	Negative
ANTI LA	< 3.3	Negative
P-ANCA	Positive	
C-ANCA	Negative	
MPO-ANCA	Positive	
Antibodies DNAn	1/160	Positive
Complement C3 (mg/dL)	89	Consumed
Complement C4 (mg/dL)	10	Consumed
Immunoglobulin *E* (UI/mL)	140	Elevated
Lambda light chain (mg/dL)	314	Elevated
Kappa light chain (mg/dL)	536	Elevated
Kappa/Lambda coefficient	1.71	Normal
Cryoglobulins	Negative	
Basal glomerular membrane antibodies	< 1/20	Negative

*Note:* Source: Patient's medical chart, 2024.

Abbreviations: ACC, anticitrulline antibodies; ANAS, antinuclear antibodies; CMV, cytomegalovirus; EB, Epstein Barr.

## Data Availability

Data sharing is not applicable to this article as no new data were created or analyzed in this study.
